# Combining S100B and Cytokines as Neuro-Inflammatory Biomarkers for Diagnosing Generalized Anxiety Disorder: A Proof-of-Concept Study Based on Machine Learning

**DOI:** 10.3389/fpsyt.2022.881241

**Published:** 2022-06-22

**Authors:** Zhongxia Shen, Lijun Cui, Shaoqi Mou, Lie Ren, Yonggui Yuan, Xinhua Shen, Gang Li

**Affiliations:** ^1^School of Medicine, Southeast University, Nanjing, China; ^2^Department of Neurosis and Psychosomatic Diseases, Huzhou Third Municipal Hospital, The Affiliated Hospital of Huzhou University, Huzhou, China; ^3^Department of Psychiatry, Wenzhou Medical University, Wenzhou, China; ^4^Department of Psychiatry, Affiliated ZhongDa Hospital of Southeast University, Nanjing, China; ^5^College of Engineering, Zhejiang Normal University, Zhejiang, China; ^6^Key Laboratory for Biomedical Engineering of Ministry of Education, Department of Biomedical Engineering, Zhejiang University, Zhejiang, China

**Keywords:** neuro-inflammatory biomarkers, S100B, cytokines, Generalized Anxiety Disorder (GAD), machine learning

## Abstract

**Introduction:**

S100 calcium-binding protein B (S100B) is a neurotrophic factor that regulates neuronal growth and plasticity by activating astrocytes and microglia through the production of cytokines involved in Generalized Anxiety Disorder (GAD). However, few studies have combined S100B and cytokines to explore their role as neuro-inflammatory biomarkers in GAD.

**Methods:**

Serum S100B and cytokines (IL-1β, IL-2, IL-4, and IL-10) of 108 untreated GAD cases and 123 healthy controls (HC) were determined by enzyme-linked immunosorbent assay (ELISA), while Hamilton Anxiety Rating Scale (HAMA) scores and Hamilton Depression Rating Scale (HAMD) scores were measured to evaluate anxiety and depression severity. This was used to help physicians identify persons having GAD. Machine learning techniques were applied for feature ordering of cytokines and S100B and the classification of persons with GAD and HC.

**Results:**

The serum S100B, IL-1β, and IL-2 levels of GAD cases were significantly lower than HC (*P* < 0.001), and the IL-4 level in persons with GAD was significantly higher than HC (*P* < 0.001). At the same time, IL-10 had no significant difference between the two groups (*P* = 0.215). The feature ranking distinguishing GAD from HC using machine learning ranked the features in the following order: IL-2, IL-1β, IL-4, S100B, and IL-10. The accuracy of S100B combined with IL-1β, IL-2, IL-4, and IL-10 in distinguishing persons with GAD from HC was 94.47 ± 2.06% using an integrated back propagation neural network based on a bagging algorithm (BPNN-Bagging).

**Conclusion:**

The serum S-100B, IL-1β, and IL-2 levels in persons with GAD were down-regulated while IL-4 was up-regulated. The combination of S100B and cytokines had a good diagnosis value in determining GAD with an accuracy of 94.47%. Machine learning was a very effective method to study neuro-inflammatory biomarkers interacting with each other and mediated by plenty of factors.

## Introduction

Generalized Anxiety Disorder (GAD) is a chronic psychiatric disease characterized by persistent, excessive worry, which seriously impairs the social and cognitive function of patients, with a lifetime prevalence of up to 6.2% in the United States (1) and 5.3% in urban China (2). Unfortunately, the diagnosis of GAD mainly depends on the syndrome, including excessive, uncontrollable worry or a disproportionate sensation of potential risk (3), all of which provide a low diagnostic accuracy. This partly resulted in the lifetime prevalence of GAD varying from 0.1% in Nigeria to 6.2% in New Zealand (4). And even within China, it varies from 0.3% (5) to 4.1–4.6% (6). Thus, far, no specific laboratory test, brain scan, or bio-marker is available to distinguish GAD from other mental disorders and healthy populations (7, 8).

Previous studies have attempted to explore the biomarkers of GAD. Genetic (7) and neurobiological biomarkers (8) failed to be applied as trait biomarkers in anxiety disorders because anxiety disorders may have different subtypes with distinctive etiopathogenesis (9). However, chronic inflammation induced by the dysfunction of the hypothalamic-pituitary-adrenal (HPA) axis in response to stressors has been identified as one of the etiologies of anxiety (10, 11). Neuro-inflammatory responses initiated by inflammatory cytokines and the subsequent neuronal dysfunction can lead to further glial activation and continued overexpression of inflammatory cytokines, which eventually cause mental disorders such as anxiety and depression (12, 13). Inflammatory biomarkers such as C reactive protein (CRP) and cytokines have been studied to identify GAD from healthy controls (HC). In their study, Tang et al. (14) reported increased CRP in GAD and a positive correlation with the severity of anxiety syndromes. Hou et al. (15) reported increased TNF-α in GAD patients compared to HC, but Vogelzangs et al. (16) found no difference between GAD and HC as well as other anxiety disorders.

Cytokines consist of pro-inflammatory cytokines such as IL-1β, IL-2, and IL-6 and anti-inflammatory cytokines such as IL-4 and IL-10 (17). Despite the fact that they are still poorly studied, the effects of these cytokines on GAD remain controversial (17). Additionally, just a limited number of studies have combined pro-inflammatory cytokines with anti-inflammatory cytokines in their research.

Peripheral cytokines are mediated by cortisol and other factors such as S100 calcium-binding protein B (S100B) and interact with each other through complex mechanisms. S100B is secreted by astrocytes in gray matter and expressed by oligodendrocytes in white matter, which modulates the proliferation and differentiation of neurons and glial cells (18). S100B is a neurotrophic factor and neuronal survival protein secreted in response to stress; S100B is always up-regulated in acute stress environments (19). A meta-analysis by Schroeter et al. (20) revealed that S100B serum levels were consistently increased in patients with depression [major depressive disorder (MDD)] and decreased after antidepressant treatment. Brown et al. (21) estimated the lifetime comorbidity of MDD and GAD as up to 90%, suggesting GAD and depression may share specific etiology and pathogenesis mechanisms. Meryem (22) reported decreased S100B levels in the hippocampus and prefrontal cortex in anxious diabetic rats, which were revised by anti-anxiety therapy. Compared to the studies on S100B levels in depression, the research on S100B as a biomarker in anxiety, especially in GAD, is limited. As a glia-originated protein, S100B activates IL-1β production through mitogen-activated protein kinase (MAPK) pathways (23). Studies have evaluated the ability of IL-8 and IL-10 combined with S100B as risk factors in alcohol use groups (24). But whether the interactions of S100B with different cytokines induce different affective diseases is still a question (25).

The neuro-inflammatory responses in GAD involve many cytokines and other factors, and cytokines are regulated by many factors and interact with each other. So, a combination of different cytokines and upstream regulators such as S100B is more likely to reveal the neuro-inflammatory responses in GAD. Existing evidence suggests that GAD patients may have abnormal serum S100B and cytokines levels, but the specific change of S100B and cytokines in GAD is still a question, and the roles of cytokines in predicting the diagnosis of GAD deserve further study. To address these lacunae, machine learning techniques for big data may help handle the multiple and asymmetrically distributed variables (26). Machine learning techniques create a paradigm shift in the prediction of diagnosis using complex computational algorithms fed by large data sets (27, 28). Recent studies have shown good prediction abilities in distinguishing patients with bipolar disorder from HC with serum biomarkers (28). We, therefore, conducted this study using machine learning algorithms to determine the roles of S100B and cytokines as neuro-inflammatory biomarkers in GAD.

## Materials and Methods

### Subjects

Patients who met the Diagnostic and Statistical Manual of Mental Disorders-IV (DSM-IV) criteria for GAD were recruited from Huzhou Third Municipal Hospital between June 2018 and June 2019. Inclusion criteria: (1) Han nationality; (2) age range 18–65 years; (3) Hamilton Rating Scale for Anxiety (HAMA) scores ≥ 17 and 17-item Hamilton Rating Scale for Depression (HAM-D_17_) ≤ 14; (4) free of major psychotropic drugs or psychotherapy for at least 4 weeks before inclusion. Exclusion criteria: (1) any mental illness requiring medical intervention such as dementia, schizophrenia, bipolar disorder, major depressive disorder, and so on; (2) substance abuse disorders; (3) inability to complete examination and questionnaires; (4) with severe physical diseases (including epilepsy, severe cardiopulmonary, malignant tumor or hematopathy, and autoimmune diseases); (5) taking immunomodulatory drugs, such as glucocorticoids, immunomodulators, antipyretic and analgesic drugs in the preceding 6 months; and (6) pregnant women. Healthy controls recruited from the local community were assessed by a psychiatrist using the Structural Clinical Interview for DSM-IV Disorders (SCID). The HAMA scores of all control subjects were ≤ 7. Those with a history of any psychiatric disorder were excluded. The protocol was approved by the Ethics Committee of Huzhou Third Municipal Hospital. Written informed consent was obtained from all participants before enrolling. Based on our pilot study, a sample size of 120 was calculated using PASS11.0 with α = 0.05 and β = 0.10. About 146 GAD patients who matched the criteria were enrolled, of which 26 refused blood tests, and blood samples from 12 patients failed to detect cytokines and S100B. According to the match ratio of 1:1, 120 HC were required, and 123 HC cases were actually enrolled.

### Rating Symptoms

The severity of the GAD symptoms was rated using HAMA, which was administered by a single trained evaluator. The HAMA entails assessing 14 items and measuring them on a 5-point scale: 0 (symptoms not present), 1 (mild symptoms), 2 (moderate symptoms), 3 (severe symptoms), and 4 (very severe symptoms). The total score is then calculated by summing the scores of the 14 items. The evaluator and laboratory staff were blind to the purpose of the study. In addition, the evaluator was blind to the laboratory data, and the HAMA scores were not disclosed to the laboratory staff. To maintain blindness, a trained research coordinator managed all data and schedules.

### Determination of Serum S100B and Cytokines

From the GAD patients, 10 ml blood samples were collected from June 2018 to June 2019 in two sterile tubes between 7 and 8 a.m. using a standard sterile preparation before treatment. Blood samples were also taken from the HC at the same time and centrifuged for 15 min at 2,500 rpm. The cell-free serum was pipetted and aliquoted in 2 ml standard freezer vials, which were then stored within 2 h at −80°C to determine the serum S100B and cytokine levels. The inflammatory cytokines, including IL-1β, IL-2, IL-4, and IL-10, were measured by ELISA. The ELISA kits were manufactured by Shanghai Yaoyun Biotechnology Limited Company. And S100B ELISA kit was manufactured by Wuhan USCN Business Co., Ltd. The sensitivity of this assay for detecting S100B, IL-1β, IL-2, IL-4, and IL-10 was 1.0 pg/L. To minimize inter-assay variations, S100B and cytokines were determined after all samples were collected.

For S100B, the inter- and intra-assay coefficients of variation were 5 and 6.5%, respectively. For cytokines, the inter- and intra-assay coefficients of variation were <10%.

### Feature Ranking and Classification

Feature ranking is of great significance in the clinical diagnosis of GAD, and important features can be extracted to assist doctors in clinical diagnosis. A feature ranking method with correlation bias reduction (RFE-CBR) based on a support vector machine (SVM) was applied to calculate the weight of all features (S100B, IL-1β, IL-2, IL-4, and IL-10). The feature with the least effect on the SVM performance was removed. The rest of the features performed the same procedure to remove the least effect feature until all the features were removed. Feature ranking was determined based on the removal order (i.e., the last deleted feature was the most important). In this study, 100 repetitions of cross-validation were performed on the training set (90% samples for the training) with the SVM-RFE-CBR method, resulting in a 100 × 5 matrix (where 5 denotes the number of features). The final ranked feature order was obtained from the 100 × 5 matrix according to the feature emerging frequency (e.g., the first important feature is the most common feature in the first column of the 100 × 5 matrix; the second important feature is the two most common features in the first two columns, and so on).

### Machine Learning Model

In this study, three popular machine learning models, SVM, random forest (RF), and integrated back propagation neural network based on bagging algorithm (BPNN-Bagging), were utilized with 10-fold cross-validation (80% for the training set and 20% samples for the testing set, respectively) (Zhang et al., 2017). Specifically, the radial basis function was applied as the kernel function for the SVM, and a decision tree number was set to 500 in the RF. As for BPNN-Bagging, 80% of features and training samples were implemented for feature perturbation and sample perturbation, and 100 BPNNs with 6 hidden layers and 100 neuron cells were used as the base learners. Then a voting method was performed on the outputs of 100 base learners to gain the final result.

### Statistical Analysis

SPSS 19.0 for Windows was used to analyze the data. Data were generally reported as mean ± SD. A Chi-square test and the independent *t*-test were performed to compare demographic data and the serum S100B/cytokines levels between GAD patients and HC. The distributions of all variables were checked by the Kolmogorov–Smirnov test, and all showed equal or nearly equal distribution. A receiver operating characteristic (ROC) curve was applied to compare the predicting value of baseline S100B and cytokines levels in GAD, Relationships between S100B/cytokines and clinical variables (age, HAMA scores, and illness duration) were evaluated using Pearson correlations.

## Results

### Demographic Information of GAD and HC

Demographic information of 108 GAD cases and 123 HC were compared, and, as shown in Table 1, there was no significant difference in the male/female ratio, age, and BMI (Body Mass Index) between the two groups. The scores of HAMA and HAMD in GAD were 22.5 ± 3.1 and 9.6 ± 2.9, respectively. The mean illness duration of GAD was 24.4 ± 37.5 months.

**Table 1:** Demographic information of GAD and HC.

**Characteristics**	**GAD**	**HC**	**χ^2^/t**	***P*** **-value**
	**(*n* = 108)**	**(*n* = 123)**		
Sex (male/female)	30/78	40/83	0.612	0.434
Age (years)	49.62 ± 11.28	47.54 ± 8.41	1.567	0.119
BMI (kg/m^2^)	21.90 ± 3.39	21.64 ± 2.99	0.620	0.536
HAMA score	22.52 ±2.99	NA		NA
HAMD score	9.63 ± 2.91	NA		NA
Illness duration (month)	24.42 ± 37.47	NA		NA

*GAD, (people with) generalized anxiety disorder; HC, healthy controls; BMI, Body Mass Index; HAMA, Hamilton Anxiety Rating Scale; HAMD, Hamilton Depression Rating Scale; NA means the value is null*.

### The ROC Value of S100B, IL-1β, IL-2, IL-4, and IL-10 in GAD

**Figure 1:**
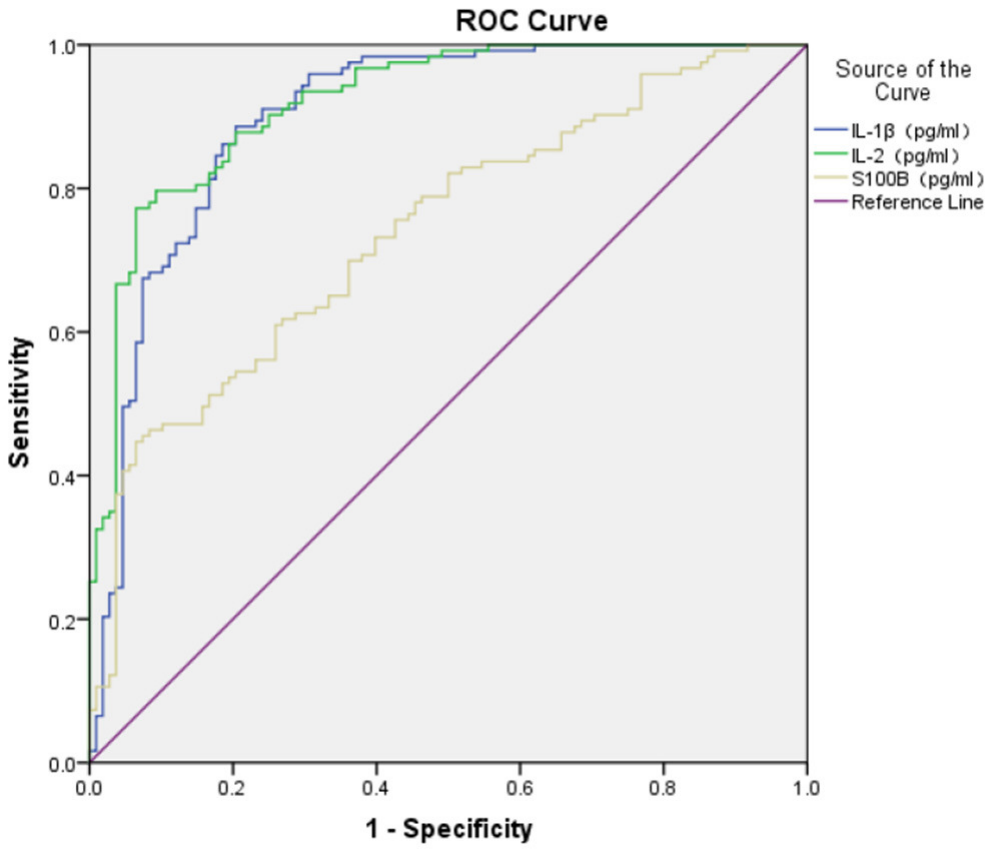
ROC of S100B, IL-1β, IL-2 in diagnosis of GAD.

**Figure 2:**
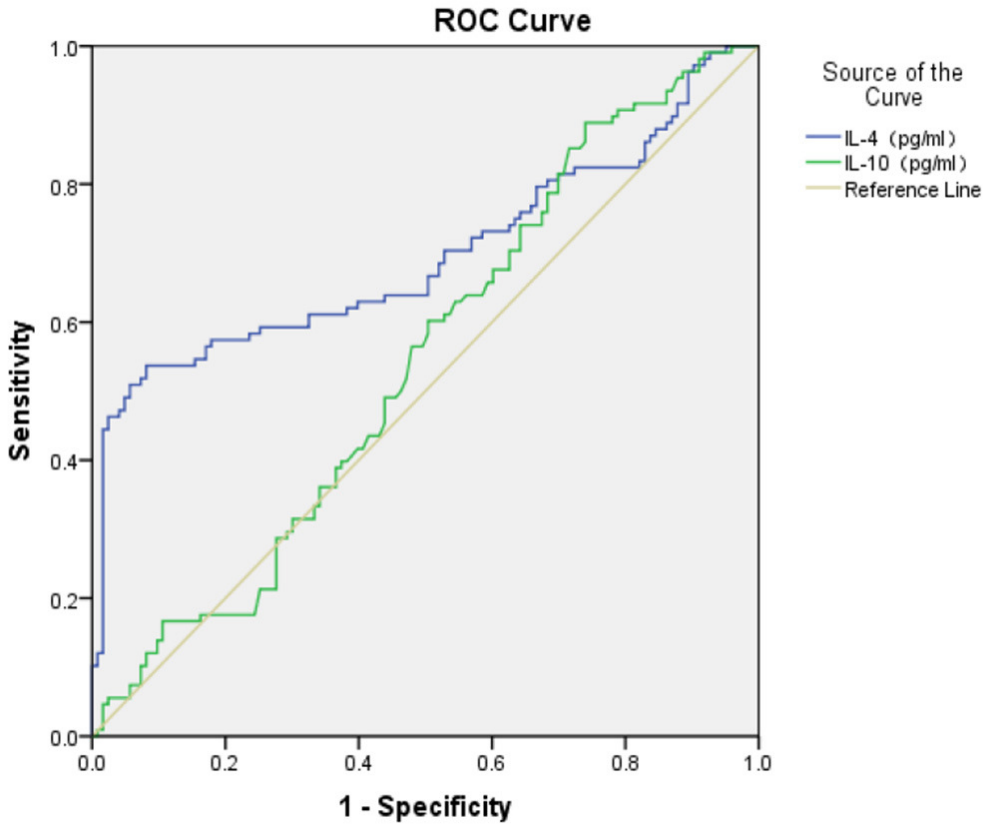
ROC of IL-4, IL-10 in diagnosis of GAD.

The ROC area of S100B, IL-1β, IL-2, and IL-4 in the diagnosis of GAD (shown in Figures 1, 2) was 0.740 ± 0.032, 0.900 ± 0.021, 0.920 ± 0.018, and 0.696 ± 0.037 respectively, and all of them suggested a good predicting value of *P* < 0.001. The ROC area of IL-10 was 0.544 ± 0.038, with a predicting value of *P* = 0.251.

### Clinical Characteristics Ranking and Statistics

**Figure 3:**
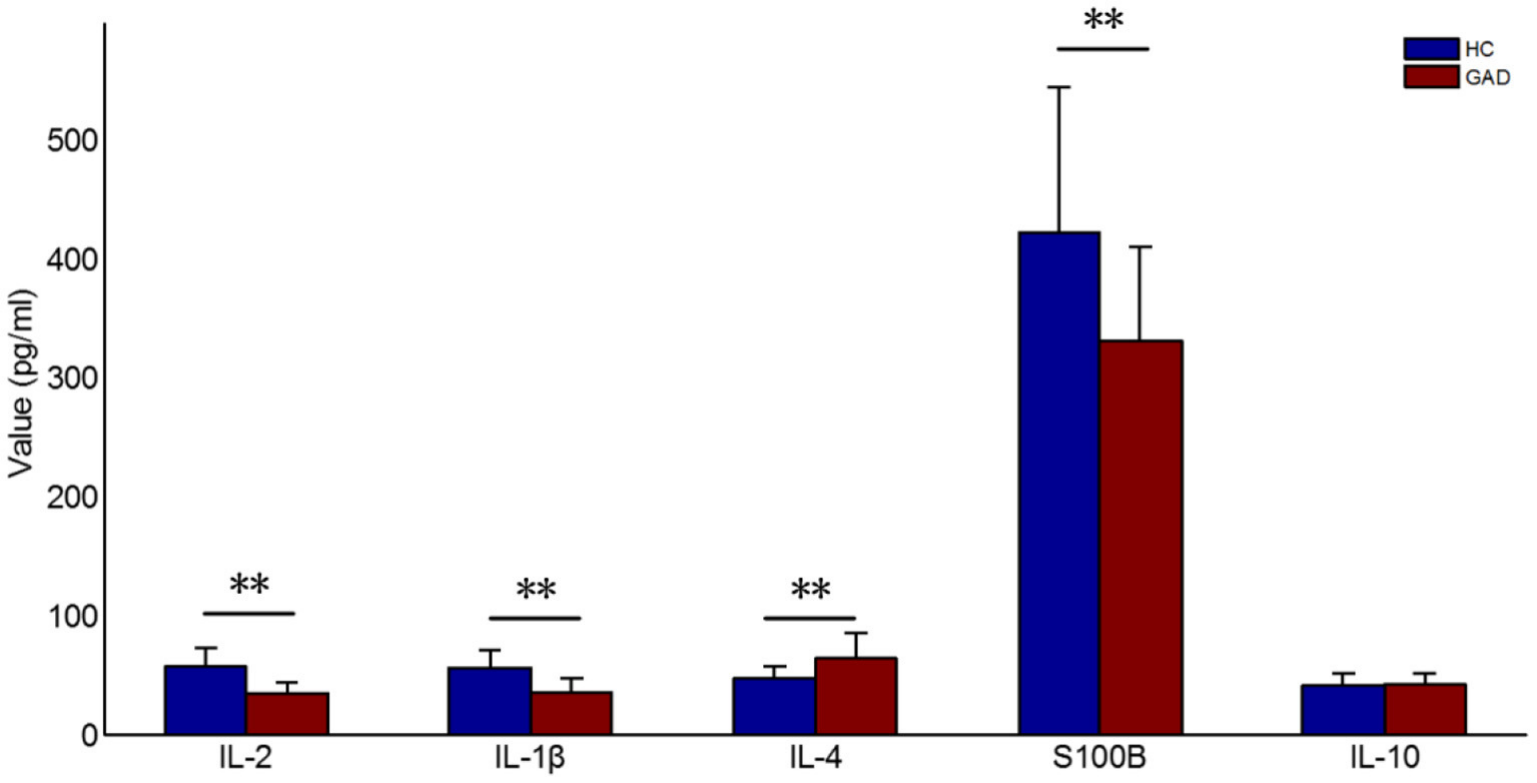
Feature ranking results of S100B, IL-1β, IL-2, IL-4, IL-10. ^**^p < 0.001.

The clinical characteristics ranking in the differential diagnosis of GAD and HC using the SVM-RFE-CBR method resulted in the following ranking order: IL-2, IL-1β, IL-4, S100B, and IL-10. As shown in Figure 3 and Table 2, the baseline serum S100B, IL-1β, and IL-2 levels of GAD cases were significantly lower than HC (*P* < 0.001), the IL-4 levels were higher than HC (*P* < 0.001), and there was no significant difference in IL-10 between the two groups (*P* = 0.215).

**Table 2:** Clinical characteristics of GAD and HC.

**Characteristics**	**GAD (*n* = 108)**	**HC (*n* = 123)**	***P*** **-value**
S100B (pg/ml)	330.28 ± 79.86	421.82 ± 122.99	<0.001
IL-1β (pg/ml)	35.52 ± 11.30	56.11 ± 14.96	<0.001
IL-2 (pg/ml)	34.68 ± 8.72	57.02 ± 16.28	<0.001
IL-4 (pg/ml)	63.71 ± 21.44	46.73 ± 10.57	<0.001
IL-10 (pg/ml)	42.65 ± 9.36	41.00 ± 10.62	0.215

### The Diagnosis of GAD Based on Machine Learning

The accuracy of S100B combined with IL-1β, IL-2, IL-4, and IL-10 in classifying GAD and HC was 91.06 ± 2.42% using SVM, 92.73 ± 3.82% using RF, and 94.47 ± 2.06% using BPNN-Bagging (Table 3).

**Table 3:** Clinical characteristics of GAD and HC.

**Model**	**SVM**	**RF**	**BPNN-bagging**
Accuracy (%)	91.06 ± 2.42	92.73 ± 3.82	94.47 ± 2.06

### Relationship Among GAD Influence Factors

The correlation of serum S100B and IL-1β, IL-2, IL-4, and IL-10 levels in GAD cases with age was *r* = −0.065, *P* = 0.505, *r* = −0.147, *P* = 0.128, *r* = −0.056, *P* = 0.568, *r* = 0.013, *P* = 0.893, and *r* = −0.113, *P* = 0.244, respectively. The correlation between serum S100B and IL-1β, IL-2, IL-4, and IL-10 was *r* = 0.175, *P* = 0.070, *r* = 0.237, *P* = 0.014, *r* = 0.261, *P* = 0.006, and *r* = 0.055, *P* = 0.589, respectively. The correlation between serum S100B and HAMA score, and illness duration was *r* = −0.386, *P* < 0.001 and *r* = 0.080, *P* = 0.410, respectively.

## Discussion

This study, to our knowledge, is the first to combine general statistical methods and machine learning to explore the characteristics of S100B and cytokines (IL-1β, IL-2, IL-4, and IL-10) in GAD patients and their role in diagnosing GAD. Currently, the exact pathology of GAD has not been well-established and is still a controversial subject. Many studies have concentrated on the neuro-inflammation pathogenesis of GAD based on the changes in cytokines as cytokines play a key role in the neuro-inflammation pathway (29, 30). Cytokines, including pro-inflammatory IL-1β, IL-2, and IL-6, and anti-inflammatory IL-4 and IL-10, are mediated by many factors such as S100B (30). S100B is mainly expressed in glial cells and plays its biological roles depending on its concentrations (19, 31–33). It acts as a growth and protection factor for neurons and astrocytes in nanomolar concentrations, and assumes neurotoxic roles, and induces the apoptosis of neurons and astrocytes in micromolar concentrations (34). The results of this study indicate that serum S100B level is significantly down-regulated in GAD patients compared to healthy controls. Few researchers have reported controversial results of S100B on anxiety. Bergh et al. (35) reported that patients with elevated S100B suffered more anxiety 3–6 years after cardiac surgery. Tomas et al. (36) found no correlation between cerebrospinal fluid S100B levels and anxiety symptoms. Another study found that S100B-positive cells and anxiety levels were markedly increased after treatment (37). But our study found that serum S100B was negatively correlated with anxiety syndromes. Buschert et al. (16) reported that elevated S100B levels increased behavioral and neural plasticity in response to acute environmental stimuli, but chronic mild stress decreased S100B in hippocampal and cerebrospinal fluid and can be revised by fluoxetine (18). S100B may be a protective factor in the acute stage of anxiety when individuals face stress. But with the development of the disease, the down-regulation of S100B may be a result of decompensation, which is closely related to the chronic pathological genesis of GAD, and the illness duration of GAD always lasts more than 6 months (chronic anxiety). S100B is mainly expressed in astrocytes and oligodendrocytes in the white matter (19), and in nanomolar concentrations, it stimulates neurite growth and increases neuronal maturation and glial cell proliferation. Reduced volumes of white matter in the dorsolateral prefrontal cortex, anterior limb of the internal capsule, and midbrain were observed in GAD patients (38). The reduced white matter volumes may help explain the down-regulation of S100B levels in GAD patients, but the interaction between the two factors needs further study.

Both central and peripheral immune dysregulation has been researched as an etiology of anxiety disorders. The results of our study reveal that GAD patients have lower serum and pro-inflammatory IL-1β and IL-2 cytokines and higher IL-4 anti-inflammatory cytokines with no dysregulated IL-10. Given the consistent hyper activation of inflammatory cytokines in depression, the results in anxiety are always controversial. Zhen Tang (14) found that serum levels of CRP, IL-1α, IL-2, IL-6, IL-8, IL-12, IFN-γ, and granulocyte-macrophage colony-stimulating factor (GM-CSF) were significantly higher in the GAD group compared to the control group, while Vogelzangs found (31) no associations with IL-6 or TNF-a in anxiety disorders including GAD. Among people with alcohol use disorder, IL-10 was negatively associated with anxiety score (39). Labaka Ainitze et al. (40) reported chronic social instability stress induced anxiety-like behavior and decreased IL-10 expression in the hippocampus of the female mice, while no differences in pro-inflammatory cytokines such as IL-1β and IL-6 were observed. Our study found decreased pro-inflammatory cytokines of IL-1β and IL-2 as well as increased anti-inflammatory cytokines IL-4, while IL-10 showed no obvious difference between GAD and control. Both cytokines and serum S100B showed a promising diagnosis value in GAD based machine learning. The classifying accuracy was 94.47 ± 2.06%. Considering the complex interaction of inflammatory mediators, cytokines in depression and other mental disorders are usually defined as networks. A single specific cytokine extends very limited influence on the whole net, but certain key cytokines may have a huge impact on a specific disorder [45]. With the help of machine learning methods, we found the feature ranking in the differential diagnosis of GAD and HC to be IL-2, IL-1β, IL-4, and IL-10. From the ranking, we can infer that IL-2 and IL-1β were key plots in the neuro-inflammation pathogenesis of GAD compared to IL-4 and IL-10, which deserve more attention in future studies.

In addition, inflammatory cytokines are influenced by many factors in anxiety disorder (30). The relation between age and cytokines has no positive connections. But it was found that serum S100B was positively correlated with IL-2 and IL-4, while it had no correlation with IL-1β and IL-10. Evidence suggests that S100B stimulates mitogen-activated protein kinase (MAPK) pathways and then induces an increase in microglial IL-1β production, and each MAPK to IL-1β production depended on the activating stimulus (23). This implies that different glial activators use distinct sets of signaling pathways to induce different inflammatory cytokines changes, which develop into different central nervous system diseases in microglia. Preliminary studies also show that S100B upregulates IL-1β and TNF-αexpression in microglia *via* the receptor for advanced glycation end products and later induces upregulation of COX-2, which eventually causes brain damage (30). Compared to IL-1β, S100B had a closer association with IL-2 in GAD in our study, though no direct evidence had been found between S100B and IL-2. Based on the results of our study, it can be inferred that the roles of S100B in the neuro-inflammation pathway of GAD may be mainly *via* the activating of IL-2 upregulation, so the downregulation of S100B coordinated with IL-2 in our work may be interpreted from this point.

There are a few limitations to our study. (1) The sample size is too small to prove that S100B's value in the neuro-inflammation pathway of GAD. (2) It would have been better to analyze the results in subgroups (sex and age), but the impact would be decreased. Samples well-matched in age and sex would be ideal for subsequent studies. (3) Although we tried to eliminate the effects of depression syndromes, GAD has high comorbidity with MDD, and stricter inclusion criteria could be helpful in future studies. (4) If we can acquire more dynamic changes in S100B and cytokines levels during the treatment (we did not collect blood samples post-intervention), the dynamic role of S100B and cytokines in the neuro-inflammation pathway of GAD will be clearer. (5) There are so many cytokines, and their inflammatory status is affected by many factors. Therefore, whether the results in this study can be duplicated in subsequent trials with other cytokines is still a question.

## Conclusion

In conclusion, our study adds to the literature by showing that serum S-100B, IL-1β, and IL-2 levels were down-regulated while IL-4 was up-regulated in persons with GAD. A combination of S100B and cytokines had a better diagnostic value with an accuracy of 94.47% than any single factor. S100B, IL-1β, IL-2, and IL-4 are very effective neuro-inflammatory biomarkers of GAD according to the clinical characteristics ranking and statistical results. There are many cytokines and regulatory factors in the neuro-inflammation pathway of GAD, and it is difficult to conclude that any specific cytokine or relevant indicator can be applied as a standard biomarker to monitor the pathological process of GAD, though limited factors were investigated in this study. Machine learning methods have been demonstrated to be very effective in studying neuro-inflammatory biomarkers interacting with each other and mediated by plenty of factors. Therefore, there are good reasons to believe that machine learning methods will play a more effective role in studying the pathological inflammatory process of GAD, which may be a network in the future compared to general statistical methods.

## Data Availability Statement

The raw data supporting the conclusions of this article will be made available by the authors, without undue reservation.

## Ethics Statement

The studies involving human participants were reviewed and approved by the Ethics Committee of Huzhou Third Municipal Hospital. The patients/participants provided their written informed consent to participate in this study. Written informed consent was obtained from the individual(s) for the publication of any potentially identifiable images or data included in this article.

## Author Contributions

Conception and design: YY and XS. Administrative support: ZS. Provision of study materials or patients: ZS and LR. Collection and assembly of data: ZS, SM, and GL. Data analysis and interpretation: LC. Manuscript writing and final approval of manuscript: All authors.

## Funding

This study was partly supported by Huzhou Public Welfare Research Project Social Development (Key Medical and Health) Category (2018GZ39, XS), Huzhou Public Welfare Research Project Social Development Category (2018GYB49, ZS), Social Development Project of Public Welfare Technology Application in Zhejiang Province in 2019 (LGF19H090003, XS), and as well as Social Development Project of Public Welfare Technology Application in Zhejiang Province in 2019 (LGF19H090002, ZS).

## Conflict of Interest

The authors declare that the research was conducted in the absence of any commercial or financial relationships that could be construed as a potential conflictof interest.

## Publisher's Note

All claims expressed in this article are solely those of the authors and do not necessarily represent those of their affiliated organizations, or those of the publisher, the editors and the reviewers. Any product that may be evaluated in this article, or claim that may be made by its manufacturer, is not guaranteed or endorsed by the publisher.
